# Effect of the COVID-19 Pandemic on Treatment Patterns, Complications and Tumour Stage in Sinonasal Malignancy Patients: A Retrospective Study

**DOI:** 10.3390/jcm15145691

**Published:** 2026-07-20

**Authors:** Joanna Cieślik, Janusz Ryś, Jerzy Tomik

**Affiliations:** 1 Doctoral School of Medical and Health Sciences, Jagiellonian University Medical College, 31-530 Krakow, Poland; 2 Department of Otolaryngology and Oncological Surgery of the Head and Neck, 5th Military Clinical Hospital with Polyclinic, 30-901 Krakow, Poland; 3 Department of Tumour Pathology, Maria Sklodowska-Curie National Research Institute of Oncology, Krakow Branch, 31-115 Krakow, Poland; janusz.rys@krakow.nio.gov.pl; 4 Department of Otolaryngology, Faculty of Medicine, Jagiellonian University Medical College, 30-688 Krakow, Poland; j.tomik@uj.edu.pl

**Keywords:** coronavirus, COVID-19 pandemic, head and neck tumour, sinonasal malignancy, TNM staging system

## Abstract

**Background:** The aim of this study was to compare tumour stage, treatment modalities, post-treatment complications, and survival among patients with sinonasal malignancies (SMs) before, during, and after the COVID-19 pandemic. **Methods:** This retrospective, two-centre study included 139 patients with SMs who were hospitalised before the COVID-19 pandemic (group 1), during the pandemic (group 2), and after the pandemic (group 3). **Results:** The study included 32, 41, and 66 patients in groups 1, 2, and 3, respectively. No significant differences were observed between the groups in terms of age, sex, recurrence rate, or the proportion of patients treated surgically. Complications were more common in group 1 than in group 2 (46.88% vs. 24.39%, *p* = 0.045). Intracranial tumour extension was observed more frequently in group 1 than in group 3 (51.61% vs. 27.27%, *p* = 0.024). The use of photon radiotherapy (RT) decreased from group 1 to group 3, whereas the use of proton RT increased and became predominant in group 3 (19.4%, 64.1%, and 95.2% in groups 1, 2, and 3, respectively). Group 3 had a higher proportion of T3 tumours than group 1 (33.3% vs. 6.3%) but a lower proportion of T4b tumours (31.8% vs. 56.3%, *p* = 0.009). Most patients had advanced disease (stage III–IV) in all groups. Kaplan–Meier survival analysis demonstrated a statistically significant improvement in survival in group 3 compared with group 1 (log-rank test, *p* = 0.048). **Conclusions:** The observed improvement is more likely attributable to shifts in tumour stage distribution and changes in treatment strategies than to any direct effect of the COVID-19 pandemic. Future research should evaluate longer post-COVID-19 periods, as potential negative effects may emerge later.

## 1. Introduction

The occurrence of malignant tumours in the nasal cavity and paranasal sinuses, accounting for 3–5% of all head and neck cancers (HNCs), classifies sinonasal malignancies (SMs) as rare neoplasms [1,2]. The mean age of patients with SM exceeds 65 years, and a higher prevalence is observed in males [2]. The most common symptoms of SM are nasal obstruction, persistent rhinorrhoea, and epistaxis. Symptoms of advanced disease are related to the involvement of surrounding structures. SMs invading the anterior cranial fossa may cause anosmia or proptosis, whereas lateral spread to the cavernous sinus can produce cranial nerve III, IV, VI, V1, and V2 neuropathies, leading to diplopia and facial paraesthesia. Mandibular paraesthesia indicates involvement of the V3 branch of the trigeminal nerve in the middle cranial fossa. Owing to the nonspecific nature of the presenting symptoms, the tumour is typically diagnosed at an advanced stage. Nowadays, the guidelines for the treatment of the most cases of SM recommend a multimodal approach combining surgery with radiation (RT), and in some cases also chemotherapy (ChT) [3]. An individualised therapeutic approach guided by a multidisciplinary tumour board is essential, taking into account the tumour stage, histopathological type, the patient’s overall clinical condition, age, and concomitant comorbidities [3].

The COVID-19 pandemic was a huge organisational challenge for all countries in the world. In accordance with pandemic-related clinical guidelines, surgical procedures for patients presenting with non-oncological diseases or conditions not deemed life-threatening were deferred. Conversely, in patients with malignancies, complications secondary to sinusitis, or cerebrospinal fluid leakage, surgical intervention should not be delayed [4]. Substantial restrictions in access to medical care caused by the high number of patients infected with the SARS-CoV-2 virus resulted in a general decrease in the number of newly diagnosed cancers [5]. Studies report a decrease in the number of the most common malignant tumours—breast, colon, and lung cancers—particularly during the first lockdown [6,7]. Data on the impact of the pandemic on patients with SMs remain limited. In a US HNC patient study, overall incidence rose 14.0% from 2020 to 2021, with the largest site-specific increase in the “sinus other” category (30.7%) [8]. Data from a cohort of 8605 HNC patients in Japan showed a decline of about 100 new cases after 2020, attributed to the impact of the COVID-19 pandemic [9]. The tumour stage of SMs was not evaluated in that study because SMs accounted for only 7% and 9% of all HNC cases in 2019 and 2020, respectively [9]. However, case reports describe patients with delayed diagnosis of SMs due to the pandemic or concomitant SARS-CoV-2 infection [6,7].

Investigations specifically addressing the incidence and stage distribution of SMs during the pandemic remain scarce [10,11,12]. Moreover, data on possible COVID-19-related sequelae and their effect on the survival of SM patients remain insufficient [4]. An observational study of 103 HNC patients showed that COVID-19-related disruption of follow-up contributed to disease progression and worse outcomes [13].

The objective of this study was to compare tumour stage at diagnosis, treatment modalities, survival, and postoperative complications among patients with SMs treated before and during the COVID-19 pandemic, and before and after the pandemic.

## 2. Materials and Methods

### 2.1. Study Design and Setting

This was a two-centre, retrospective cohort study conducted at the Department of Otolaryngology and Oncological Surgery of the Head and Neck, 5th Military Hospital (a tertiary care hospital) and at the Maria Skłodowska-Curie National Research Institute of Oncology, Kracow, Poland. During the first lockdown, surgical treatment was continued, with priority given to patients with HNC or life-threatening conditions. The study conducted in accordance with the Declaration of Helsinki and was approved by the Jagiellonian University Bioethical Committee (No. 1072.6120.329.2021).

### 2.2. Participants

We reviewed electronic and paper medical records of patients hospitalised for SMs before the COVID-19 pandemic (1 January 2017–20 March 2020; group 1), during the pandemic (21 March 2020–13 May 2022; group 2), and after the pandemic (14 May 2022–31 December 202; group 3). Patients with SMs were identified using ICD-10 codes ‘C30’ and ‘C31.1-31.3’, ‘C31.9-31.9’, according to the 10th revision of the International Classification of Diseases (ICD-10).

Each patient was assigned exclusively to a single group. The inclusion criteria were newly diagnosed or recurrent SMs. Patients with ear tumours (identified by ICD-10 code C30.9 were excluded because of the small number of cases in the entire cohort (*n* = 11) and because their inclusion would have increased the heterogeneity of the study population.

### 2.3. Variable

Demographic characteristics (age, sex), admission date, duration of symptoms before the first appointment, type of treatment (surgery, radiotherapy (RT), ChT or combinations of the previously mentioned), surgical approach, tumour characteristics (location, presence of nodal and distant metastases, intracranial extension), time interval between surgery and initiation of RT, doses used for RT, post-treatment complications, histopathological work-up, and follow-up data were recorded. The TNM stage was assessed according to the Union for International Cancer Control’s 8th edition of the TNM Classification of Malignant Tumours. Complications such as mucositis and dermatitis were described according to the Radiation Therapy Oncology Group (RTOG) scale of The European Organisation for Research and Treatment of Cancer (EORTC). To assess temporal variations related to COVID-19, we compared group 1 with group 2 (pre- vs. intra-pandemic) and group 1 with group 3 (pre- vs. post-pandemic). Mortality incidence was assessed on 16 February 2026.

### 2.4. Statistical Analysis

Qualitative variables were expressed as counts (percentages) and compared using Pearson’s Chi-square test, or Fisher’s exact test when at least 20% of contingency table cells had an expected count below 5. The Shapiro–Wilk test assessed the normality of continuous variables. Because age was non-normally distributed, the Mann–Whitney U test was used and values were reported as medians with quartiles. Because RT duration was also non-normally distributed, the non-parametric Kruskal–Wallis test was used. For ordinal variables, including TNM classification and cancer stage, the differences between groups were assessed using the Fisher–Freeman–Halton test. The Kaplan–Meier method and the log-rank test were employed to compare differences in time from treatment initiation to death or the end of follow-up. The threshold for statistical significance was set at *p* < 0.05, except where adjusted using the Bonferroni correction. Statistical analyses were conducted using PS IMAGO PRO 9.0 software (Predictive Solutions Sp. z o.o., Krakow, Poland), incorporating the IBM SPSS Statistics 29.0 analytical engine (IBM Corp., Armonk, NY, USA).

## 3. Results

### 3.1. Baseline Characteristics

The study included a cohort of 139 patients, with 32, 41, and 66 individuals in groups 1, 2, and 3, respectively. There were no significant differences in sex or age between the groups. The median age was 58 years in group 1, 56 years in group 2, and 61 years in group 3. The median duration of symptoms before the first appointment was 5, 4, and 5 months in groups 1, 2, and 3, respectively. Performance status, accessed on the Eastern Cooperative Oncology group Performance Status Scale (ECOG), was equal to 1 in most patients in each group. Cancer recurrence rates did not differ significantly between the groups, and approximately one-third of patients in each group had a recurrence (Table 1). There was a higher percentage of patients in the intra-pandemic group than in the pre-pandemic group with a history of prior RT (24.4% vs. 6.3%, *p* = 0.038), and no differences were found between post- and pre-pandemic groups (*p* = 0.211). Regarding post-treatment complications, these were observed more frequently in group 1 than in group 2 (46.88% vs. 24.39%, *p* = 0.045). The most common complications were grade 2 or 3 mucositis and grade 2 or 3 dermatitis; one patient in group 1 died during the proton therapy period.

### 3.2. Tumour Characteristics

There were no significant differences in tumour localisation within the paranasal sinuses when comparing group 1 with group 2 or group 1 with group 3. However, tumours in both groups 2 and 3 involved both sides of the paranasal sinuses. Interestingly, intracranial tumour extension was most frequent in group 1 (51.61%), and the difference between group 1 and group 3 (27.27%) reached statistical significance (*p* = 0.024). The rates of nodal involvement and distant metastasis were low across all groups, with no significant differences detected (Table 2).

### 3.3. Treatment Modality

Surgery served as the treatment for the majority of patients, comprising 65.6%, 75.6% and 69.7% in groups 1, 2 and 3, respectively (Table 1).

Table 3 shows the distribution of surgical management strategies across the groups, including an endoscopic approach, craniotomy, hybrid surgery (endoscopic plus craniotomy), an open approach (lateral rhinotomy or a sublabial approach), and diagnostic biopsy only. Open approach was the most common overall, especially in group 2 (43.24%) and group 1 (38.71%), and was less frequent in group 3 (28.57%). Biopsy-only procedures were also frequent and showed similar rates groups. Endoscopic approaches predominated in group 3 (31.75%) compared with group 2 (16.22%) and group 1 (12.90%). Craniotomy was infrequent and limited to group 1 (9.68%) and group 2 (8.11%), with no such procedure in group 3. Hybrid approaches (endoscopic plus craniotomy) were rare but more frequent in group 3 (7.94%) than in group 2 (5.41%) and group 1 (3.23%).

Table 4 illustrates the distribution of RT decisions across the groups. The use of photon RT decreased markedly from group 1 to group 3, whereas the use of proton RT increased substantially, becoming the predominant treatment in group 3 (19.4%, 64.1%, and 95.2% in group 1, 2, and 3, respectively). Decisions to omit RT (due to tumour extent and the risk of fatal side effects) and patient-initiated treatment discontinuation remained uncommon in all groups.

The median duration of RT was the highest in group 3 (median 66.5 days, Q1 = 42.8, Q3 = 74.3); for groups 1 and 2 the median was equal to 50 days (for group 1 Q1 = 34, Q3 = 54.5; for group 2 Q1 = 46, Q3 = 71 days). A significant difference was observed between groups 1 and 3 (Kruskal–Wallis test with Bonferroni correction, *p* = 0.016). The interval between surgery and RT was comparable across the three groups, with median (and Q1; Q3) times of 117 (111; 124), 119 (78; 153), and 91 (57; 126) days for groups 1, 2, and 3, respectively (Figure S1).

Regarding RT, 25, 33, and 58 patients in groups 1, 2, and 3, respectively, received RT at one of our hospitals. The median dose to the main tumour area was similar in groups 1 and 3, at 70 Gray (Gy), whereas it was 66 Gy in group 2 (Table S1). The median dose to areas at high risk of recurrence was 63 Gy in each group. Among patients eligible for RT, not all received RT to the lymph node region (Table S1). Specifically, 14, 20, and 36 patients in groups 1, 2, and 3, respectively, underwent lymph node RT.

### 3.4. Histopathological Work-Up

Squamous cell carcinoma (SCC) was the most common diagnosis in all groups, accounting for 21.9% in group 1, 24.4% in group 2, and 33.3% in group 3 (Figure S2). Olfactory neuroblastoma (ONB) represented 21.9% of tumours in group 1 and 18.2% in group 3, while its frequency in group 2 was lower (9.8%). Adenoid cystic carcinoma (ACC) was also frequently encountered, representing 18.8% of cases in group 1, 19.5% in group 3, and 7.6% in group 2. Sinonasal mucosal melanoma was identified in 12.5% of group 2, 9.1% of group 1, and 7.6% of group 3. Sinonasal undifferentiated carcinoma (SNUC) constituted 14.6% of group 2, 9.4% of group 1, and 9.1% of group 3 (Figure S2). For the main histopathological subtypes (SCC, ACC, ONB, adenocarcinoma, and sinonasal melanoma), the distribution of subtypes was similar across groups.

### 3.5. TNM Classification

Regarding tumour stage, the analysis showed a higher prevalence of T4a or T4b disease in groups 1 and 2 than in group 3 (Table 5). In group 3, approximately one-third of the patients were in each of the T3, T4a or T4b stages (33.3%, 25.8%, and 31.8%, respectively). There was a statistically significant difference in the distribution of T classification between group 1 and group 3 after Bonferroni correction (*p* = 0.009; corrected significance level: *p* < 0.025). Group 3 exhibited a higher proportion of T3 tumours (33.3%) compared with group 1 (6.3%), whereas the proportion of T4b tumours was lower in group 3 (31.8%) than in group 1 (56.3%). No differences were found between groups 1 and 2 with respect to T and N categories (*p* = 0.567 and *p* = 0.117, respectively). In all groups, more than 80% of cases had no lymph node metastases, and more than 96% had no distant metastases (Table 5).

### 3.6. Stage

In the entire cohort, most of the patients had advanced-stage disease (stage III–IV), accounting for 97%, 90%, and 91% in groups 1, 2, and 3, respectively (Figure S3). The number of patients increased markedly in advanced stages (III–IVB), particularly in group 3, which showed the highest counts across most stages (Figure S3). Stage IVB represented the most frequent stage in all groups (53.1%, 42.5%, and 31.8%, in groups 1, 2, and 3, respectively). There was no statistically significant difference in the distribution of tumour stages between group 1 and group 3 (Fisher–Freeman–Halton test, *p* = 0.056) or between groups 1 and 2 (*p* = 0.831, Figure S3).

### 3.7. Survival Analysis

In the follow-up analysis of the entire cohort, 53 patients (38.1%) had died by February 15, 2026. Eleven patients were lost to follow-up (two patients in group 2, five patients in group 2, and four patients in group 3). Among patients who died, the median survival time from the end of treatment to death was 16 months (95% confidential interval, [CI] 12.0; 30.4) in group 1, 14.5 months (95% CI 10.5; 26.2) in group 2, and 9 months (95%CI 8.0; 16.8) in group 3, with no significant differences between the analysed groups (the Kruskal–Wallis test: groups 1 vs. 2, *p* = 0.875; groups 1 vs. 3, *p* = 0.207).

**Figure 1:**
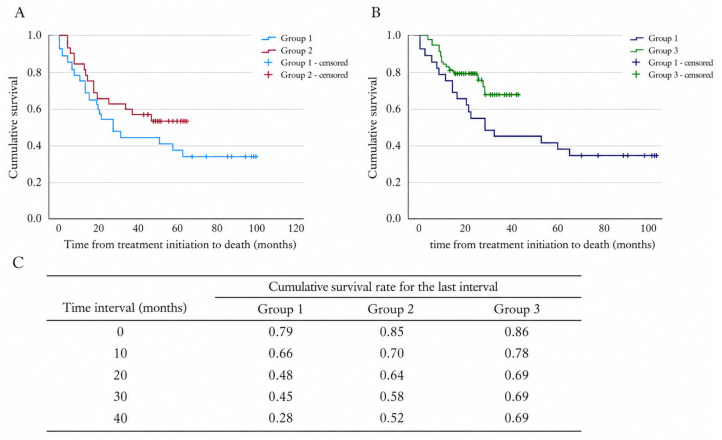
Kaplan–Meier curves for overall survival, defined as the time from treatment initiation to death, comparing group 1 with group 2 (**A**) and group 1 with group 3 (**B**). Cumulative survival rates by group are shown in (**C**). Log-rank test: A, *p* = 0.217; B, *p* = 0.048.

Kaplan–Meier analysis showed no difference in time from treatment completion to death between groups 1 and 2. (log-rank test, *p* = 0.217, Figure 1A). In contrast, a significant improvement in survival was observed in group 3 compared with group 1 (log-rank test, *p* = 0.048; Figure 1B). The cumulative survival rate at 40 months after treatment completion was 28% in group 1 and 69% in group 3.

The overall survival rates at 12 months after treatment completion were 55%, 67%, and 78% for groups 1, 2, and 3, respectively. At 36 months after treatment completion, the corresponding overall survival rates were 28%, 50%, and 64%. The median survival time for group 1 was 10.4 months (95% CI: 10.4–45.6), whereas the median survival was not reached for groups 2 and 3.

## 4. Discussion

To our knowledge, this is the first study to compare treatment modalities, types of surgery, complications, and tumour stage in patients with SMs not only before and during the COVID-19 pandemic but also before and after the pandemic. SM often presents with nonspecific symptoms that overlap significantly with benign inflammatory conditions or SARS-CoV-2 infection, which could potentially have contributed to diagnostic delays during the COVID-19 pandemic. Furthermore, the good general condition of patients with SMs, also observed in our cohort, usually does not prompt immediate medical attention. However, SMs are relatively rapidly growing tumours that develop in an anatomically and functionally complex area, making prompt treatment crucial [14].

Our study showed slight differences in the selection of surgical strategies between groups. In the intra-pandemic group, open surgical approaches were the most common, whereas the post-pandemic group showed greater use of endoscopic and hybrid techniques, and the pre-pandemic group had a more balanced distribution of procedures. The choice of surgical approach was related to tumour advancement, tumour location, intracranial extension, and the surgeon’s experience [3,15]. It is well-established that the surgical approach is not the primary factor associated with patient survival. Rather, the most significant determinants include histopathological tumour type, advanced patient age, higher tumour stage, the presence of nodal metastasis [16], and clear resection margins [17,18]. On the other hand, endoscopic sinus and skull base surgeries were considered aerosol-generating procedures [19]. Therefore, at the beginning of the pandemic, open surgery was recommended, as it was associated with a lower risk of infection for the staff [20]. Over time, new studies have emerged on aerosol generation during endoscopic surgery [21]. High-speed drilling and cauterization generated substantial aerosols, while nasal suction and cutting forceps did not produce aerosols at detectable levels [22]. Recommendations on microdebrider use were inconsistent [21].

Due to the proximity of vital organs at risk, including the brain, brainstem, eyes, and cranial nerves, it is imperative to strictly limit the radiation dose [23]. In proton therapy, the beam energy can be delivered to a precise depth, resulting in reduced toxicity compared with conventional photon RT. In a subgroup analysis of the meta-analysis comparing proton beam therapy with intensity-modulated radiation RT (IMRT), proton therapy was associated with significantly improved 5-year disease-free survival (relative risk (RR) 1.44, 95% CI 1.01–2.05; *p* = 0.045) and superior locoregional control at the longest available follow-up (RR 1.26, 95% CI 1.05–1.51; *p* = 0.011) [24]. An investigation conducted across nine European RT hubs, encompassing multiple oncological sites, identified delays in cancer diagnosis during the COVID-19 pandemic that may have contributed to patients presenting with more advanced stages of disease at the time of symptom onset [25]. In our study, the time from surgery to RT was similar between the analysed groups. Furthermore, that study [25] favoured a hypofractionated regimen, in contrast to our study, which employed conventional fractionation, with an identical number of fractions administered in all groups. Regarding RT modalities, an increase in proton RT was noted in four centres in that study, whereas a stable trend was observed in the other institutions [25]. In our cohort, we observed a clear shift from photon to proton RT across the groups. Considering that most of our patients had advanced SM, the choice of proton RT was dictated by the need to limit side effects. In the pandemic group, more patients had undergone prior irradiation than in the pre-pandemic group, which may also have influenced the decision to use proton rather than photon RT. We hypothesise that the pandemic did not significantly influence this process; instead, it was mainly affected by increased proton therapy availability and growing clinical experience with this modality among radiation oncologists.

Among all histopathological subtypes, SCC is the most prevalent, followed by adenocarcinoma in the second position [3,26]. The variety and rarity of histopathological types of SM can make diagnosis challenging. It is highly recommended to seek the second opinion of a well-experienced pathologist, due to the discrepancy between histopathological diagnoses discrepancies in histopathological diagnosis are not uncommon and can radically alter management [3,27]. Our findings demonstrate a heterogeneous distribution of tumour histological subtypes, with SCC, ONB, ACC, and SNUC representing the most prevalent entities within the study cohort, with no apparent impact of the pandemic on this distribution.

In our cohort, stage III tumours were more frequent in the post-pandemic group than in the pre-pandemic group (33.3% vs. 6.3%), whereas the distribution of other stages was similar between groups. Most patients in each group had advanced-stage disease. A Swiss study comparing the pre-pandemic and pandemic periods demonstrated that the total number of newly diagnosed SM cases in 2020 was comparable to that observed during the two preceding years [10]. Moreover, during the pandemic period, a higher percentage of advanced-stage cases was observed, although this difference was not statistically significant (36.7% vs. 63.2%, *p* = 0.064) [10]. A nationwide study from the Netherlands reported similar percentages of patients in 2018, 2019, and 2020 years [12]. However, that study evaluated stage among all HNC patients and did not separately distinguish patients with SM. Most studies on the effects of the pandemic on patients with HNC did not consider patients with SM separately because of the small number of cases [28,29].

This study did not identify statistically significant differences in overall survival between patients treated before and during the COVID-19 pandemic, suggesting the absence of a detectable pandemic-related decline in prognosis within this cohort. In contrast, patients treated in the post-pandemic period demonstrated superior survival outcomes compared with those treated before the pandemic. These observed differences may be explained by variations in tumour stage, more frequent intracranial tumour extension, and higher rates of post-treatment complications in the pre-pandemic group than in the post-pandemic group.

### Limitations of the Study

These results should be interpreted cautiously, given the study’s retrospective design and small sample size. However, this must be considered in the context of SM as a rare disease. In addition, we included multiple histopathological tumour subtypes, each characterised by distinct patterns of biological behaviour, response to treatment, and survival; however, the distribution of subtypes was comparable between the groups.

## 5. Conclusions

Our study shows that pandemic-related changes in healthcare did not increase cancer stage at diagnosis in patients with SM. Future research on the consequences of the COVID-19 pandemic conditions should include multicentre cohorts of patients with SM. This experience highlights the urgent need to ensure uninterrupted access to essential cancer care during global crises.

## Figures and Tables

**Table 1:** Baseline characteristics. Data are given as number (percentage in group). Q1—the first quartile; Q3—the third quartile. ECOG scale: Eastern Cooperative Oncology Group scale; Pearson’s Chi-square test χ^2^ was used unless otherwise stated. ^ The Mann–Whitney test was used. The results are significant at *p* < 0.05.

Characteristics		Group 1 *n* = 32	Group 2 *n* = 41	Group 3 *n* = 66	*p*-Value (1 vs. 2)	*p*-Value (1 vs. 3)
Age, median (Q1; Q3), years		58 (43; 64)	56 (44; 66)	61 (49; 69)	0.565 ^	0.192 ^
Age (min; max), years		16; 80	26; 84	18; 81		
Men sex		17 (53.1)	24 (58.5)	40 (60.6)	0.644	0.481
Duration of symptoms, months	median (Q1; Q3),	5 (1.75; 12.0)	4 (2.5; 6.0)	5 (2.0; 10.5)	0.688	0.857
	min; max	0; 36	1; 24	1; 132		
Recurrence		12 (37.5)	16 (39.0)	18 (27.3)	0.894	0.303
Performance status (ECOG)	0	0	3 (7.3)	2 (3.0)	0.204	0.610
	1	31 (96.9)	35 (85.4)	62 (93.9)		
	2	1 (3.1)	3 (7.3)	2 (3.0)		
Prior radiation		2 (6.3)	10 (24.4)	11 (16.7)	0.038	0.211
Surgical treatment		21 (65.63)	31 (75.61)	46 (69.70)	0.350	0.684
Chemotherapy		12 (37.50)	11 (26.83)	23 (34.85)	0.330	0.797
Post-treatment complications		15 (46.88)	10 (24.39)	39 (59.09)	0.045	0.254

**Table 2:** Tumour characteristics. Data are given as number (percentage in group); Pearson’s Chi-square test χ^2^ was used unless otherwise stated. * Fisher’s exact test was used. ^&^ No data for two patients. NA: not assessed.

Tumour Characteristics		Group 1 *n* = 32	Group 2 *n* = 41	Group 3 *n* = 66	*p*-Value (1 vs. 2)	*p*-Value (1 vs. 3)
Location						
	left	13 (40.63)	24 (58.54)	17 (25.76)	0.304	0.251
	right	12 (27.50)	10 (24.39)	26 (39.39)		
	both	7 (21.88)	7 (17.07)	23 (34.85)		
Intracranial extension ^&^		16 (51.61)	15 (37.50)	18 (27.27)	0.234	0.024
Nodal metastasis		6 (18.75)	4 (9.76)	10 (15.38)	0.317 *	0.675
Distant metastasis		1 (3.33)	1 (2.78)	2 (3.33)	NA	NA

**Table 3:** Type of surgery approach in groups. The percentages (in groups) refer to the groups of patients who underwent surgical treatment. Fisher’s exact test was used. A significance threshold of 0.025 was applied according to the Bonferroni correction.

Surgery Approach	Group 1 *n* = 32	Group 2 *n* = 41	Group 3 *n* = 66	*p*-Value (1 vs. 2)	*p*-Value (1 vs. 3)
Endoscopic approach	4 (12.90)	6 (16.22)	20 (31.75)	0.748	0.339
Craniotomy	3 (9.68)	3 (8.11)	0		
Hybrid approach (endoscopic + craniotomy)	1 (3.23)	2 (5.41)	5 (7.94)		
Open approach	12 (38.71)	16 (43.24)	18 (28.57)		
Biopsy only	11 (35.48)	10 (27.03)	20 (31.75)		

**Table 4:** Data are given as number (percentage). Missing data were observed for one, two and four patients in groups 1, 2, and 3, respectively. RT, radiotherapy. Fisher’s exact test was used for pairwise comparisons of group 1 vs. group 2 and group 1 vs. group 3. Superscript letters denote statistically significant differences from group 1 after Bonferroni correction; group 2 and group 3 were not compared. The Bonferroni-corrected significance level was set at *p* < 0.025.

RT	Group 1 *n* = 32	Group 2 *n* = 41	Group 3 *n* = 66	*p*-Value (1 vs. 2)	*p*-Value (1 vs. 3)
photon RT	19 (61.3) ^a^	9 (23.1) ^b^	1 (1.6) ^b^	<0.001	<0.001
proton RT	6 (19.4) ^a^	25 (64.1) ^b^	59 (95.2) ^b^		
No RT, physician’s decision	6 (19.4) ^a^	4 (10.3) ^a^	1 (1.6) ^a^		
RT discontinuation, patient’s decision	0 ^a^	1 (2.6) ^a^	1 (1.6) ^a^		

**Table 5:** TNM (Tumour, Node, Metastasis) classification in groups, based on the Union for International Cancer Control TNM Classification of Malignant Tumours, 8th edition. Data are presented as number (percentage). The Fisher–Freeman–Halton test was used separately for T, N, and M classifications to compare group 1 vs. group 2 and group 1 vs. group 3. The Bonferroni-corrected significance level was set at *p* < 0.025. A statistically significant difference in the distribution of T classification was found between group 1 and group 3 (*p* = 0.009), whereas no significant differences were found for N or M classification.

		Group 1 *n*= 32	Group 2 *n* = 41	Group 3 *n* = 66	*p*-Value (1 vs. 2)	*p*-Value (1 vs. 3)
T	1	0	0	3 (4.5)		
	2	1 (3.1)	4 (10.0)	3 (4.5)	0.567	0.009
	3	2 (6.3)	3 (7.5)	22 (33.3)		
	4a	11 (34.4)	16 (40.0)	17 (25.8)		
	4b	18 (56.3)	17 (42.5)	21 (31.8)		
N	0	27 (84.4)	37 (92.5)	56 (84.8)		
	1	1 (3.1)	3 (7.5)	5 (7.6)		
	2a	2 (6.3)	0	1 (1.5)	0.117	0.248
	2b	0	0	3 (4.5)		
	2c	2 (6.3)	0	1(1.5)		
M	0	29 (97.7)	35 (97.2)	58 (96.7)	0.706	0.709
	1	1 (3.3)	1 (2.8)	2 (3.3)		

## Data Availability

The datasets used and/or analysed during the current study are available from the corresponding author on reasonable request.

## Supplementary Materials

The following supporting information can be downloaded at https://www.mdpi.com/article/10.3390/jcm15145691/s1, Figure S1. Time interval between surgery and initiation of radiotherapy in groups. The Kruskal-Wallis test for comparison between groups 1 and 2 *p* = 0.919, between groups 1 and 3 *p* = 0.074. Figure S2. Histopathological outcomes in groups. Data are given as percentage in the group. SNUC, Sinonasal undifferentiated carcinoma. Figure S3. Cancer stage in groups. The Fisher–Freeman–Halton exact test was used, *p*-value between groups 1 vs. 2 and 1 vs. 3 were 0.831 and 0.056, respectively. Table S1. Doses used for radiotherapy in groups for patients who received RT in the Maria Skłodowska-Curie National Research Institute of Oncology, Cracow, Poland.

## Abbreviations

The following abbreviations are used in this manuscript:

SM	Sinonasal Malignancy
HNC	head and neck cancers
ECOG	Eastern Cooperative Oncology group Performance Status Scale
RT	Radiotherapy
ChT	Chemotherapy
SCC	Squamous cell carcinoma
ONB	Olfactory Neuroblastoma
ACC	Adenoid cystic carcinoma
SNUC	Sinonasal undifferentiated carcinoma
TNM	Tumour Node Metastasis Classification
