# Supplementary material for: Effect of the COVID-19 Pandemic on Treatment Patterns, Complications and Tumour Stage in Sinonasal Malignancy Patients: A Retrospective Study
Source: J Clin Med. 2026 Jul 20;15(14):5691. doi: 10.3390/jcm15145691 (PMC13413089; doi:10.3390/jcm15145691)
Supplement: Supplementary file 1 [file jcm-15-05691-s001.zip › jcm-4377736-supplementary.pdf]

Supplementary materials

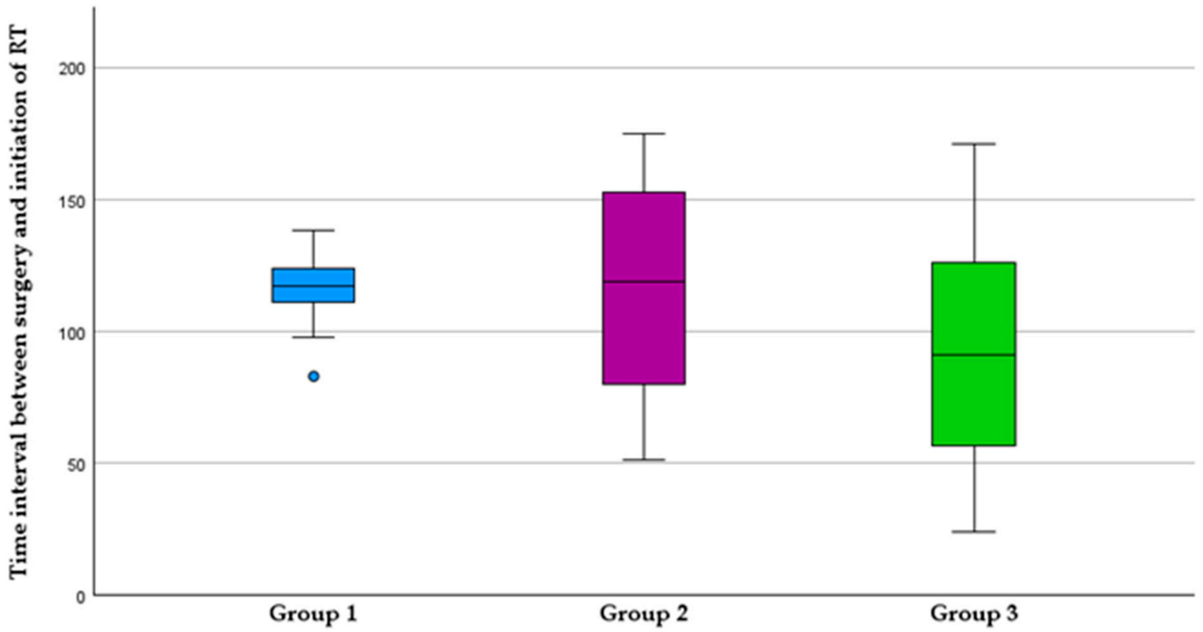

Figure S1. Time interval between surgery and initiation of radiotherapy in groups. The Kruskal-Wallis test for comparison between groups 1 and 2 $p = 0.919$, between groups 1 and 3 $p = 0.074$.

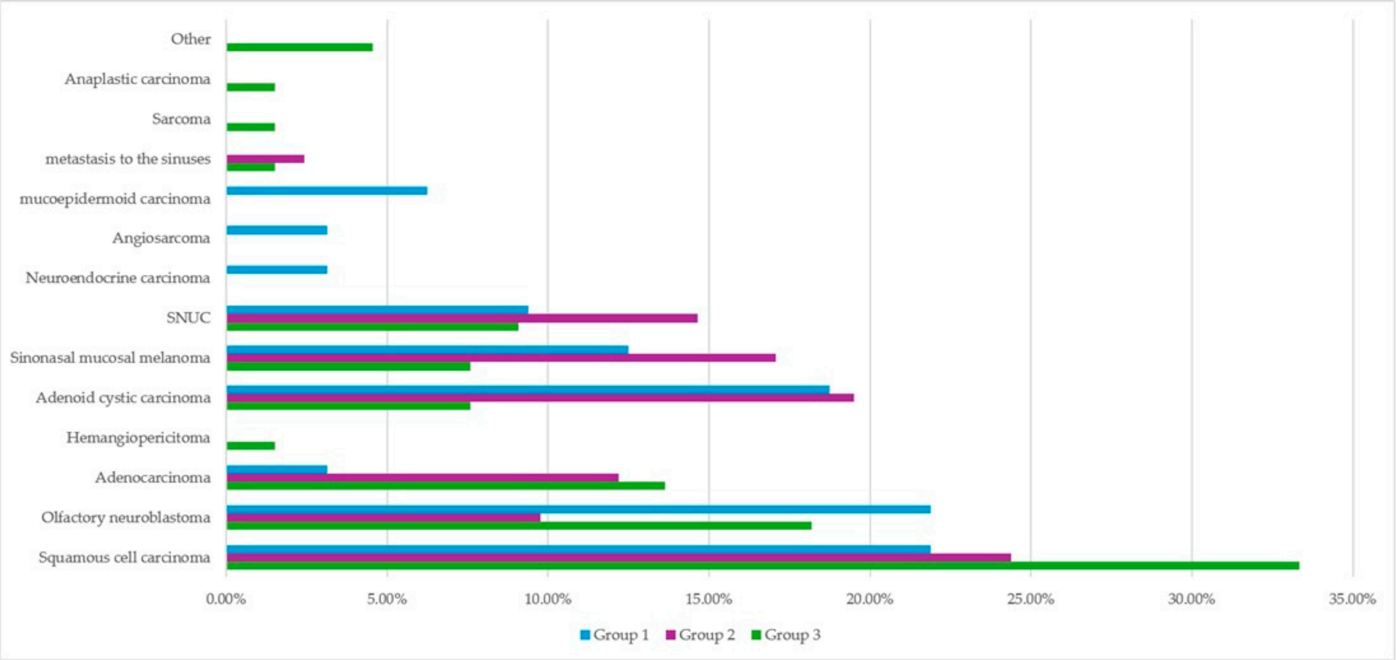

Figure S2. Histopathological outcomes in groups. Data are given as percentage in the group. SNUC, Sinonasal undifferentiated carcinoma.

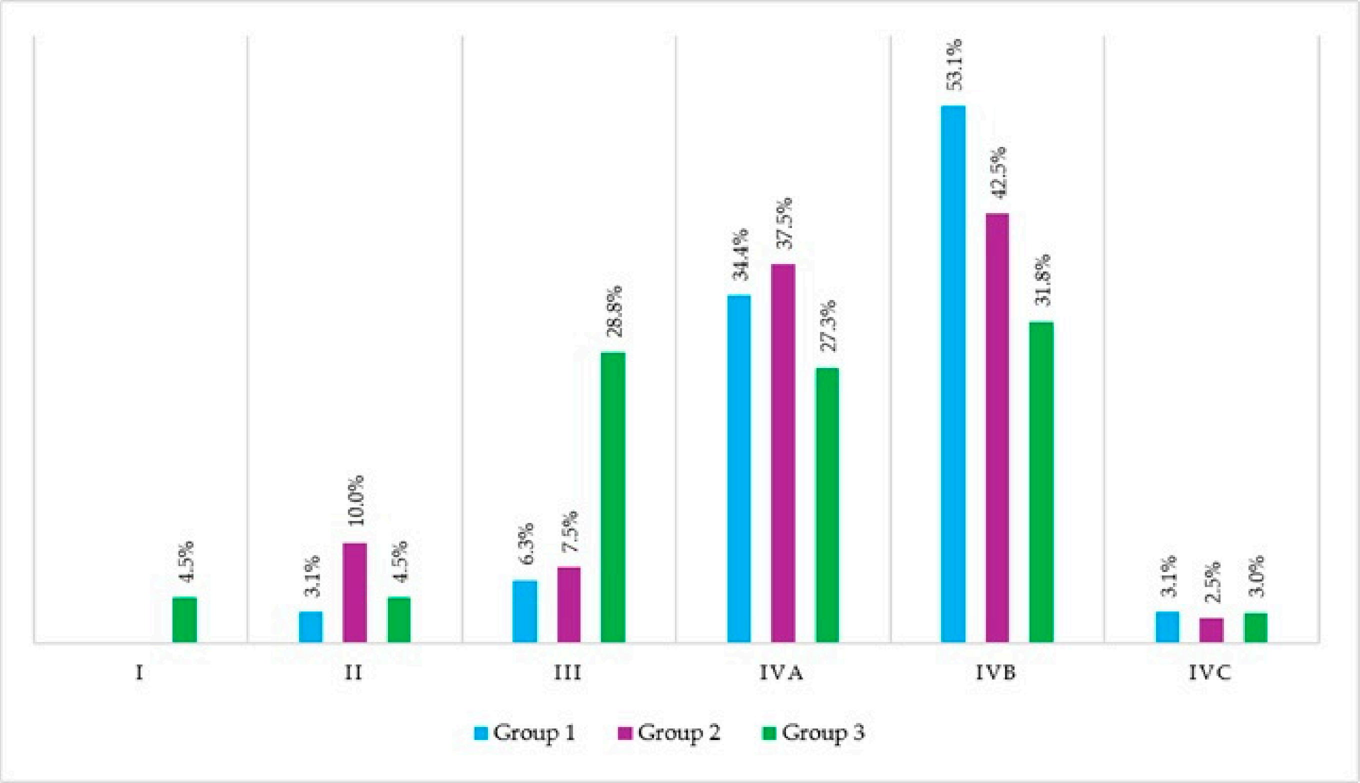

Figure S3. Cancer stage in groups. The Fisher–Freeman–Halton exact test was used, *p*-Value between groups 1 vs 2 and 1 vs 3 were 0.831 and 0.056, respectively.

Table S1. Doses used for radiotherapy in groups for patients who received RT in the Maria Skłodowska-Curie National Research Institute of Oncology, Cracow, Poland.

		**Group 1**	**Group 2**	**Group 3**	***p*-Value (1 vs. 2)***	***p*-Value (1 vs. 3)***
		n=25	*n*=33	*n*=58		
the main tumor area	dose [Gy]	70 (54.9; 70)	66 (61.4; 70)	70 (66; 70)	0.787	0.142
	number of fractions	35 (30; 35)	33 (30; 35)	35 (33; 35)	0.728	0.155
		n=14	*n*=22	*n*=47		
the high-risk area	dose [Gy]	63 (30; 63)	63 (60; 65)	63 (60; 66)	0.143	0.083
	number of fractions	33 (15; 35)	33 (30; 35)	33 (30; 35)	0.924	0.850
		n=14	*n*=20	*n*=36		
lymph node area	dose [Gy]	30 (25; 35)	54 (54; 56)	54 (54;56)	0.486	0.050
	number of fractions	30 (25; 35)	31 (28; 35)	30 (30; 35)	0.241	0.167

Data are given as median (quartile 1 and quartile 3) in the group. Gy - Gray. * Kruskal-Wallis test was used.
